# Peri‐Implantitis and Survival Outcomes of Tissue Level Versus Bone Level Dental Implants: A Systematic Review and Meta‐Analysis

**DOI:** 10.1002/cre2.70242

**Published:** 2025-10-16

**Authors:** Momen A. Atieh, Maanas Shah, Abeer Hakam, Ahmad Aid, Andrew Tawse‐Smith, Nabeel H. M. Alsabeeha

**Affiliations:** ^1^ Hamdan Bin Mohammed College of Dental Medicine Mohammed Bin Rashid University of Medicine and Health Sciences Dubai United Arab Emirates; ^2^ Faculty of Dentistry, Sir John Walsh Research Institute University of Otago Dunedin New Zealand; ^3^ School of Dentistry University of Jordan Amman Jordan; ^4^ College of Dentistry Ajman University Ajman United Arab Emirates

**Keywords:** dental implants, meta‐analysis, osseointegration, peri‐implantitis, systematic review

## Abstract

**Objectives:**

The aim of this systematic review and meta‐analyses was to evaluate the outcomes of using tissue level in comparison to bone level implants in terms of clinical and radiographic changes, peri‐implantitis, and implant failure rates.

**Materials and Methods:**

Electronic databases were searched to identify randomized studies that compared tissue level to bone level implant. The risk of bias was assessed using the Cochrane Collaboration's Risk of Bias tool. Data were analyzed using a statistical software program.

**Results:**

A total of 798 studies were identified, of which, five studies with 501 dental implants in 241 participants were included. Overall meta‐analysis showed that the use of tissue level implants had lower rates of peri‐implantitis (relative risk [RR] 0.59; 95% confidence interval [CI] 0.14–2.48; *p* = 0.47) and implant failure (RR 0.59; 95% CI 0.07–4.69; *p* = 0.62) but the differences were not statistically significant.

**Conclusions:**

Tissue level and bone level have comparable survival rates and risk of peri‐implantitis within 1–5 years of observation. Additionally, no significant differences in probing pocket depths and marginal bone level changes were observed.

**Clinical Relevance:**

Tissue level implants demonstrated lower rates of peri‐implantitis and implant failure, as well as smaller changes in probing pocket depths at 12 and 24 months, compared to bone level implants. Although these differences were not statistically significant, they suggest potential clinical advantages of tissue level implants in maintaining peri‐implant health and stability over time.

## 1 Introduction

The long‐term survival rate of dental implants can be adversely affected by peri‐implant diseases, a bacterially‐induced inflammation of the peri‐implant tissues (Borges et al. 2020; Fernandes et al. 2022; Fernandes et al. 2024; Serino et al. 2023). A mean incidence rate of 18.8% for peri‐implant mucositis and 9.6% for peri‐implantitis has been reported (Atieh et al. 2022a, 2013, 2019; Heitz‐Mayfield et al. 2020). Peri‐implant diseases could be influenced by various implant and prosthetic design elements such as restorative contour and angle, implant‐abutment connection, implant to crown ratio, and the location of the implant‐abutment interface, be it at the level of bone or soft tissues (Atieh et al. 2010, 2023; Dixon and London 2019; Hamilton et al. 2023; Mattheos et al. 2021; Lin et al. 2025; Janda and Mattheos 2024; Puisys et al. 2023). A tissue level implant has an integral transmucosal component that positions the implant‐abutment interface at the soft tissue level, away from the bone. In contrast, bone level implants have their implant‐abutment interface located at the crest of the bone. Comparable survival and esthetic outcomes have been demonstrated for tissue level and bone level implants in short‐term studies (Astrand et al. 2004; Lago et al. 2019; Liu et al. 2021; Sanz‐Martín et al. 2017; Siebert et al. 2018; Wallner et al. 2018; Lombardi et al. 2025). However, when peri‐implant diseases are considered, the outcome seems to be less conclusive. For example, Mattheos et al. (2021) conducted a review on six primary studies comparing peri‐implant disease prevalence in tissue level and bone level implants. In one study (Katafuchi et al. 2018), bone level implants have shown higher prevalence compared to tissue level implants (22.8% vs. 7.5%, respectively), but the statistical significance was not detected. No significant difference in the prevalence of peri‐implantitis between bone level implants of external and internal connection and tissue level implants (29.8%, 17.5%, and 13.6%, respectively) was reported by Laleman and Lambert (2023), however, only 6% of the implants tested were tissue level.

Despite numerous studies and reviews comparing tissue level and bone level implants (Astrand et al. 2004; Hadzik et al. 2017; Lago et al. 2019; Liu et al. 2021; Mortazavi et al. 2021; Sanz‐Martín et al. 2017; Wallner et al. 2018), there remains a lack of conclusive evidence from randomized controlled trials on outcomes of tissue level and bone level implants of similar surface characteristics. Therefore, the purpose of this systematic review and meta‐analysis was to evaluate the clinical and radiographic outcomes of tissue level implants in comparison to bone level implants as reported in randomized controlled trials.

## 2 Materials and Methods

The guidelines of the Cochrane Collaboration and Preferred Reporting Items for Systematic Reviews and Meta‐analyses (Page et al. 2021) were followed in the development of the current systematic review. Based on the participant, intervention, comparison, outcomes, and study design (PICOS) framework (Higgins et al. 2024; Richardson et al. 1995), the following criteria were established:

Participant: Human adults aged ≥ 18 years who require replacing missing teeth with dental implants.

Intervention: Tissue level implant.

Comparison: Bone level implant.

Outcomes: Peri‐implantitis rate, changes in marginal bone level, changes in probing pocket depth, and implant failure rate.

Study design: Randomized studies of interventions.

The study was registered with the National Institute for Health Research under the PROSPERO ID CRD42024582587. Ethical approval was not required for this systematic review.

### 2.1 Study Selection and Criteria

This review included randomized studies comparing tissue level and bone level dental implants with similar surface characteristics. Eligible studies were required to report on marginal bone levels (whether showing changes or stability), peri‐implantitis or implant failure rates. No restrictions were applied regarding language or publication status. Non‐randomized studies, case series, case reports, histomorphometric research, and studies that did not provide sufficient data were excluded.

### 2.2 Participants and Interventions

Participants that were 18 years of age or older and required either tissue level or bone level dental implants to replace missing teeth. The intervention group involved the replacement of missing teeth with tissue level implants, while the control group involved the replacement of missing teeth with bone level implants. Any implant placement or loading protocol was considered (Hammerle et al. 2004; Gallucci et al. 2018).

### 2.3 Outcome Measures

Primary outcome: Peri‐implantitis rate.

Secondary outcomes: Changes in marginal bone level, changes in probing pocket depth, and implant failure rate.

### 2.4 Search Strategy

The search protocol followed accepted methodological guidance for systematic reviews (Faggion and Park 2013; Higgins et al. 2023). We systematically searched MEDLINE (via PubMed), EMBASE, Cochrane Central Register of Controlled Trials (CENTRAL), Web of Science, Scopus, Google Scholar, and ClinicalTrials.gov up to September 26, 2025 (Table 1). The searches were performed independently and in duplicate by two reviewers (M.A. and N.A.). To ensure comprehensive retrieval, we used a combination of controlled vocabulary (Medical Subject Headings [MeSH] in MEDLINE; Emtree terms in EMBASE) and free‐text keywords. The use of free‐text terms was necessary because some recent trials, abstracts, or unpublished studies may not yet be indexed with controlled vocabulary, and certain implant terminology (e.g., “tissue‐level,” “one‐piece,” and “transmucosal”) is not consistently captured by MeSH terms. Equivalent strategies were adapted for each database.

**Table 1:** Databases and search terms.

Database	Search terms (MeSH and free‐text)
Published studies	
PubMed (MEDLINE) (up to September 26, 2025)	(“dental implants”[MeSH] OR “dental implantation”[MeSH] OR “dental implant*” OR “oral implant*”) AND (“tissue level” OR “one piece” OR “transmucosal”) AND (“bone level” OR “two piece”) AND (“clinical trial” OR “randomized controlled trial”)
EMBASE (up to September 26, 2025)	(‘dental implant’/exp OR ‘oral implant’/exp OR ‘dental implant’:ti, ab OR ‘oral implant’:ti, ab) AND (“tissue level”:ti, ab OR “one piece”:ti, ab OR “transmucosal”:ti, ab) AND (“bone level”:ti, ab OR “two piece”:ti, ab) AND (‘clinical trial’/exp OR ‘randomized controlled trial’/exp OR “clinical trial”:ti, ab OR “andomized controlled trial”:ti, ab)
Cochrane Central Register of Controlled Trials (CENTRAL) (up to September 26, 2025)	(“dental implant*” OR “oral implant*”) AND (“tissue level” OR “one piece” OR “transmucosal”) AND (“bone level” OR “two piece”)
Web of Science (up to September 26, 2025)	TS = (“dental implant*” OR “oral implant*”) AND TS = (“tissue level” OR “one piece” OR “transmucosal”) AND TS = (“bone level” OR “two piece”)
Scopus (up to September 26, 2025)	TITLE‐ABS‐KEY (“dental implant*” OR “oral implant*”) AND TITLE‐ABS‐KEY (“tissue level” OR “one piece” OR “transmucosal”) AND TITLE‐ABS‐KEY (“bone level” OR “two piece”)
Google scholar (up to September 26, 2025)	“dental implant” AND “tissue level” AND “bone level” AND “randomized trial”
Unpublished studies	
ClinicalTrials.gov (up to September 26, 2025)	(“dental implant*” OR “oral implant*”) AND (“tissue level” OR “one piece” OR “transmucosal”) AND (“bone level” OR “two piece”)

Abbreviation: MeSH, medical subject headings.

In addition, we screened the bibliographies of all eligible full‐texts and performed a manual search of the last 5 years of pertinent journals (*Clinical and Experimental Dental Research, Clinical Implant Dentistry and Related Research*, *Clinical Oral Implants Research*, *International Journal of Oral and Maxillofacial Implants*, *International Journal of Periodontics and Restorative Dentistry*, and *Journal of Periodontology*). This process was adapted from our previous systematic reviews (Atieh et al. 2025, 2024a, 2024c, 2024b, 2022b).

### 2.5 Selection of Studies

The titles, abstracts, and keywords of the retrieved citations were screened separately and in duplicate by two reviewers (M.A. and N.A.). After eliminating irrelevant papers, the full texts of the remaining ones were collected. An eligibility form was used to assess potential papers for inclusion in the review. Disagreements between reviewers were resolved through discussions or by consulting a third reviewer (M.S.). When duplicate papers were selected, the one with the most adequate and relevant information was chosen. Reasons for exclusion were mentioned.

### 2.6 Data Collection

Using a data extraction form, two reviewers (M.A. and N.A.) separately gathered the following information from the included studies: (1) Study characteristics: Title, authors’ names, study location, language of publication, year of publication, published or unpublished data, source of study funding, study design (parallel group or split mouth), and method of randomization, allocation concealment, and blinding (participants, investigators, and outcome examiners). (2) Participants: Demographic characteristics, inclusion/exclusion criteria, number of participants in test and control groups, attrition rate, and reasons for dropouts. (3) Interventions: Number of participants where tissue level dental implants were placed. (4) Comparison: Number of participants where bone level dental implants were placed. (5) Outcomes: Peri‐implantitis and implant failure rates, changes in marginal bone level and probing pocket depth. (6) Length of the observation period. Any differences of opinion amongst reviewers were settled via consensus‐building discussions or by consulting a third reviewer (M.S.). Corresponding authors of included studies were contacted when additional information was required.

### 2.7 Quality Assessment of Included Studies

Two reviewers (M.A. and N.A.) evaluated the risk of bias for each of the included studies separately and in duplicate. The randomized studies were evaluated using the Cochrane risk of bias tool (Higgins et al. 2023).

### 2.8 Data Synthesis

A statistical program (Review Manager [RevMan] software, version 5.3, The Nordic Cochrane Center, The Cochrane Collaboration, Copenhagen, Denmark) was used to perform meta‐analyses for studies of similar comparisons reporting the same end measures. Continuous data, such as changes in the marginal bone level, was expressed as mean difference (MD) or standardized mean difference and 95% confidence intervals (CIs). Since study heterogeneity was anticipated, the results from multiple studies were pooled using the random‐effects model. Split‐mouth and parallel group studies were combined using the generic inverse variance option in the statistical software program.

Because the power to detect publication bias was low (less than 10 papers), publication bias was not officially assessed (Higgins et al. 2024). The Cochran's test for heterogeneity and *I* ^2^ statistic were used to evaluate the statistical heterogeneity between various studies (Higgins et al. 2024). Significant heterogeneity was indicated by an *I* ^2^ score greater than 60. The implant served as the analysis’ statistical unit. To investigate the cause of heterogeneity, the stability of the results, and the impact of the studies, a leave‐one study‐out sensitivity analysis was carried out. Sensitivity analysis was used to find out whether estimated effects changed when analyses that included studies with a high risk of bias were omitted. The GRADE criteria (risk of bias, inconsistency, imprecision, indirectness, and publication bias) were used to evaluate the certainty of evidence (Higgins et al. 2024). A software program (GRADEpro Guideline Development Tool software, McMaster University and Evidence Prime, 2021, available from gradepro.com) was used to create summary of findings table.

## 3 Results

### 3.1 Characteristics of the Study Settings

**Figure 1:**
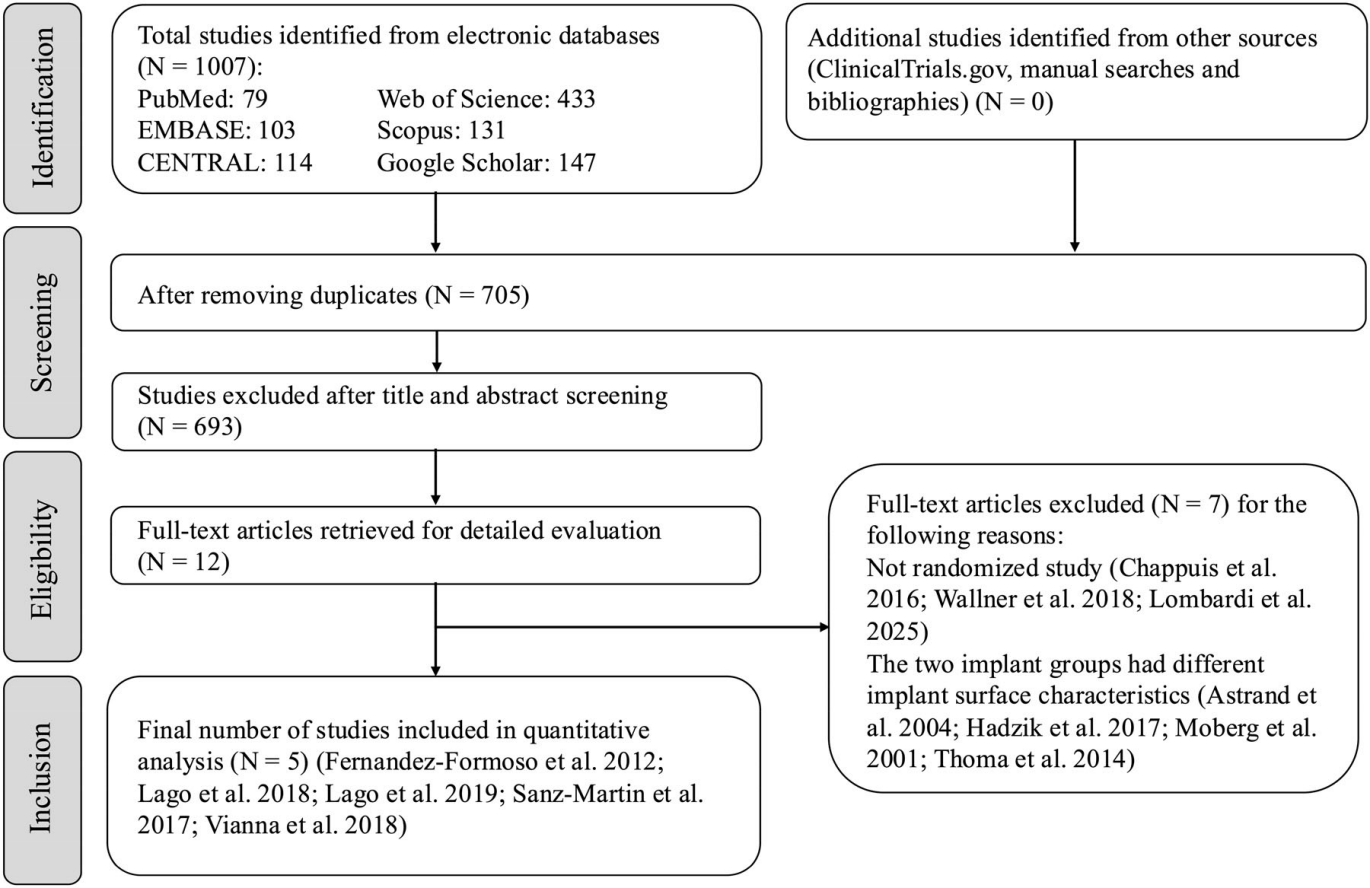
Flowchart of the search process.

A total of 1007 studies were initially retrieved from the databases (Figure 1). After independent and duplicate screening of titles and abstracts by two reviewers (M.A. and N.A.), 12 studies were selected for full‐text review (Astrand et al. 2004; Chappuis et al. 2016; Fernández‐Formoso et al. 2012; Hadzik et al. 2017; Lago et al. 2018, 2019; Moberg et al. 2001; Sanz‐Martín et al. 2017; Thoma et al. 2014; Vianna et al. 2018; Wallner et al. 2018; Lombardi et al. 2025). Of these, three (Chappuis et al. 2016; Wallner et al. 2018; Lombardi et al. 2025) were excluded for not being randomized controlled trials and four (Astrand et al. 2004; Hadzik et al. 2017; Moberg et al. 2001; Thoma et al. 2014) were excluded because they compared implant designs with different surface characteristics. Consequently, five randomized controlled trials (Fernández‐Formoso et al. 2012; Lago et al. 2018, 2019; Sanz‐Martín et al. 2017; Vianna et al. 2018) were included in the present review (Table 2). Four of these were conducted in Spain (Fernández‐Formoso et al. 2012; Lago et al. 2018; Lago et al. 2019; Sanz‐Martín et al. 2017), and one in Brazil (Vianna et al. 2018). Three (Fernández‐Formoso et al. 2012; Lago et al. 2018; Sanz‐Martín et al. 2017) had a parallel‐group design, and two (Lago et al. 2019; Vianna et al. 2018) employed a split mouth approach. One study (Sanz‐Martín et al. 2017) reported industry funding, whereas the remaining four (Fernández‐Formoso et al. 2012; Lago et al. 2018, 2019; Vianna et al. 2018) were self‐funded. All were carried out in university settings.

**Table 2:** Characteristics of the included studies.

	**Fernández‐Formoso et al. (** 2012 **)**	Lago et al. (2018)	Lago et al. (2019)	Sanz‐Martín et al. (2017)	Vianna et al. (2018)
Study design	RCT (parallel group)	RCT (parallel group)	RCT (split mouth)	RCT (parallel group)	RCT (split‐mouth)
Location	University of Santiago de Compostela, Santiago de Compostela, Spain	University of Santiago de Compostela, Santiago de Compostela, Spain	University of Santiago de Compostela, Santiago de Compostela, Spain	University Complutense of Madrid, Spain	State University of Campinas, Piracicaba, Sao Paulo, Brazil
Number evaluated (participants/implants)	51/114	98/200	35/100	37/47	20/40
TLI	25/56	50/100	35/50	19/25	20/20
BLI	26/58	48/100	35/50	18/22	20/20
Age (years)	43.29 ± 10.20	50.44 ± 10.89	49.5 ± 11.25	58.70 ± 11.15	49.13 ± 6.76
Smoking habits					
TLI	0	0	NR	6	0
BLI	0	0	NR	4	0
Implant system					
TLI	*	*	*	‡	*
BLI	†	†	†	§	†
Implant diameter (mm)	3.3, 4.1, 4.8	3.3, 4.1, 4.8	NR	3.8, 4.25, 5	NR
Implant height (mm)	8–14	8–12	NR	7–13	NR
Implant placement protocol	Type IV	Type IV	Type IV	Type IV	Type IV
	One‐stage surgical technique	One‐stage surgical technique	One‐stage surgical technique	One‐stage surgical technique	One‐stage surgical technique
Implant loading protocol	Type C	Type C	Type C	Type C	Type C
Implant location	Mandible and maxilla	Mandible and maxilla	Mandible and maxilla	Mandible and maxilla	Mandible and maxilla
Method of assessment	Periapical digital radiographs using a standardized paralleling technique	Periapical radiographs using long‐cone paralleling technique	Manual periodontal probe	Manual periodontal probe^I^	Manual periodontal probe^I^
			Periapical radiographs using long‐cone paralleling technique	Standardized digital periapical radiographs	Peri‐apical radiographs with individualized acrylic occlusal stents
Changes in MBL (mm) at 12 months					
TLI	0.42 ± 0.11	0.26 ± 0.55	0.15 ± 0.49	−0.27 ± 0.24	NR
BLI	0.04 ± 0.50	−0.03 ± 0.74	0.08 ± 0.26	−0.12 ± 0.19	NR
Changes in MBL (mm) at 24 months					
TLI	NR	NR	NR	NR	0.75 ± 1.12
BLI	NR	NR	NR	NR	0.70 ± 0.72
Changes in MBL (mm) at 36 months					
TLI	NR	NR	0.18 ± 0.46	NR	NR
BLI	NR	NR	0.14 ± 0.35	NR	NR
Changes in MBL (mm) at 60 months					
TLI	NR	0.61 ± 0.73	NR	NR	NR
BLI	NR	−0.20 ± 0.75	NR	NR	NR
Changes in PPD (mm) at 12 months					
TLI	NR	NR	2.10 ± 0.60	3.17 ± 0.70	2.79 ± 0.46
BLI	NR	NR	2.20 ± 0.50	2.95 ± 0.70	3.08 ± 0.68
Changes in PPD (mm) at 24 months					
TLI	NR	NR	NR	NR	3.39 ± 0.63
BLI	NR	NR	NR	NR	3.52 ± 0.58
Peri‐implantitis N(%)					
TLI	0 (0.0)	0 (0.0)	0 (0.0)	25 (8.0)	0 (0.0)
BLI	0 (0.0)	1 (1.0)	0 (0.0)	22 (9.1)	1 (5.0)
Implant failure N(%)					
TLI	0 (0.0)	0 (0)	0 (0.0)	0 (0.0)	0 (0.0)
BLI	0 (0.0)	1 (1.0)	0 (0.0)	1 (4.5)	0 (0.0)
Follow‐up period (months)	12	60	36	12	24

Abbreviations: BLI, bone level implant; MBL, marginal bone level; NR, not reported; PPD, probing pocket depth; RCT, randomized controlled trial; TLI, tissue level implant.

*Standard plus, Institut Straumann AG, Waldenburg, Switzerland.

^†^Bone level, Institut Straumann AG, Waldenburg, Switzerland.

^‡^One‐piece premium TG, Sweden and Martina implants, Due Carrare, Padova, Italy.

^§^Two‐piece premium SP, Sweden and Martina implants, Due Carrare, Padova, Italy.

^I^ PCP UNC 15; HuFriedy, Chicago, IL, USA.

### 3.2 Participant Characteristics

Across the five trials, participants were generally healthy adults requiring replacement of single or multiple missing teeth in the anterior or posterior regions of the maxilla or mandible. The minimum age varied from 20 to 35 years, while some studies specified ranges up to 70 years. Most trials required adequate bone dimensions (types II or III), sufficient keratinized tissue, good plaque control, and a minimum healing period of 6 months following tooth extraction. In some cases, additional requirements included neighboring teeth adjacent to the edentulous site and opposing natural dentition or implant‐supported prostheses.

Exclusion criteria were consistent across studies and aimed at eliminating factors that could compromise osseointegration or long‐term implant outcomes. Patients with uncontrolled systemic diseases, immunodeficiencies, prior implant surgery in the treated area, untreated periodontitis, temporomandibular disorders, bruxism, history of radiation or chemotherapy, or substance abuse were excluded. Pregnant or lactating women and individuals with mucosal diseases were also not eligible. Smoking was generally an exclusion factor, though thresholds varied between studies.

### 3.3 Interventions

Preoperative management included appropriate implant planning using cone beam computed tomography, models, and diagnostic wax‐up (Vianna et al. 2018; Lago et al. 2018, 2019). Before implant surgery, intraoral antisepsis was carried out utilizing a 0.12% chlorhexidine solution and 4 mg of dexamethasone was administered as an anti‐inflammatory treatment (Vianna et al. 2018). Antibiotics were used 1 h before surgery and for 8 days after surgery (Lago et al. 2018, 2019). Implant surgeries were performed according to manufacturers’ instructions with the transmucosal machined collar of tissue level implants placed at the level of soft tissue, while bone‐level implants were placed at the crestal bone level (Fernández‐Formoso et al. 2012; Lago et al. 2018, 2019; Sanz‐Martín et al. 2017). The countersink drill was solely used in the tissue level implant group (Sanz‐Martín et al. 2017). A resorbable membrane and xenograft bone substitute were used in the event of implant dehiscence or fenestrations (Sanz‐Martín et al. 2017). In split‐mouth studies, the implant osteotomies were prepared simultaneously and the opaque sealed envelopes were opened to identify implant design just before using the profile and thread drills (Vianna et al. 2018). The implants were placed following a one‐stage surgical protocol (Vianna et al. 2018). A healing abutment was placed and interrupted sutures were used to close the flap (Lago et al. 2018, 2019; Vianna et al. 2018). The final prosthesis was delivered between 2 (Lago et al. 2018, 2019) and 12 months (Sanz‐Martín et al. 2017) after implant placement and torqued to 40 Ncm (Sanz‐Martín et al. 2017). Standardized digital periapical radiographs and baseline clinical measurements were recorded at implant placement, prosthesis installation, 12 months post‐loading, and at each follow‐up visit (Fernández‐Formoso et al. 2012; Lago et al. 2018, 2019; Sanz‐Martín et al. 2017).

Postoperative care involved mouth rinsing with 0.12% chlorhexidine (Sanz‐Martín et al. 2017; Vianna et al. 2018) and brushing the treated area with a surgical brush (Sanz‐Martín et al. 2017). In addition, anti‐inflammatory medications were prescribed as needed by the patient (Sanz‐Martín et al. 2017). Participants were enrolled in a supportive therapy program following implant placement with monthly visits for the first 9 months of follow‐up and every 3 months for the next 24 months following the delivery of implant‐supported prosthesis (Vianna et al. 2018). The follow‐up time varied between 12 (Fernández‐Formoso et al. 2012; Sanz‐Martín et al. 2017) and 60 months (Lago et al. 2018).

### 3.4 Outcome Measures

The primary outcome across all trials was the incidence of peri‐implantitis (Lago et al. 2018; Sanz‐Martín et al. 2017; Vianna et al. 2018; Fernández‐Formoso et al. 2012; Lago et al. 2019). Secondary outcomes included marginal bone level changes (Fernández‐Formoso et al. 2012; Lago et al. 2018, 2019; Sanz‐Martín et al. 2017; Vianna et al. 2018), probing pocket depth (Lago et al. 2019; Sanz‐Martín et al. 2017; Vianna et al. 2018), and implant failure rate (Fernández‐Formoso et al. 2012; Lago et al. 2018, 2019; Sanz‐Martín et al. 2017; Vianna et al. 2018). Marginal bone levels were evaluated using standardized periapical radiographs, and probing pocket depths were measured clinically by periodontal probe.

### 3.5 Risk of Bias in Randomized Controlled Trials

**Figure 2:**
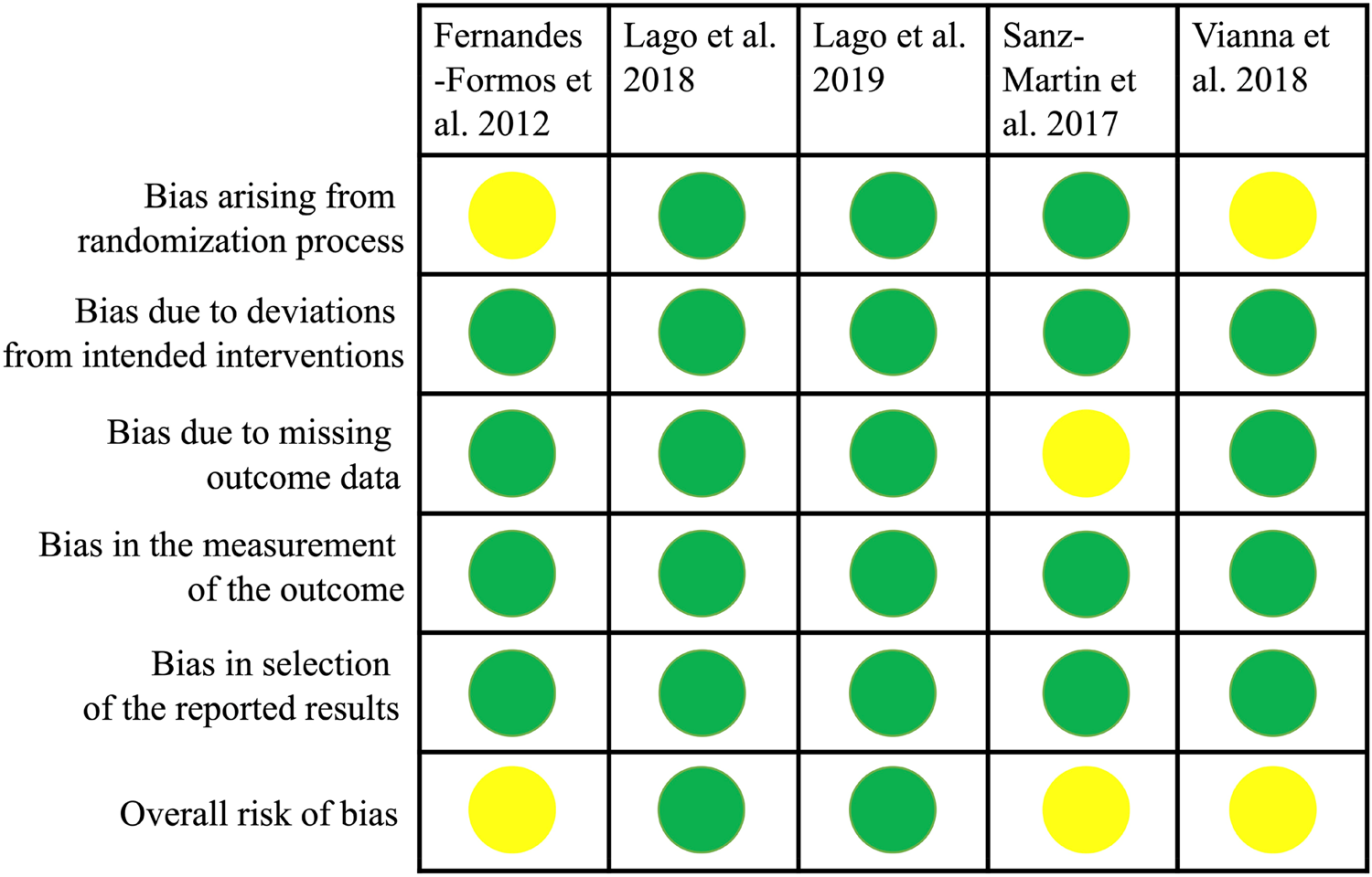
Assessment of risk of bias of the randomized studies presented with low (green), some concerns (yellow), and high (red) risk of bias.

All the included studies were randomized controlled trials. Overall, two studies (Lago et al. 2018, 2019) were judged to be at low risk of bias, while the remaining three (Fernández‐Formoso et al. 2012; Sanz‐Martín et al. 2017; Vianna et al. 2018) were graded at some risk of bias concerns (Table 3, Figure 2).

**Table 3:** Assessment of risk of bias of the included randomized controlled trials.

	F**erná**ndez‐Formoso et al. (2012)	Lago et al. (2018)	Lago et al. (2019)	Sanz‐Martín et al. (2017)	Vianna et al. (2018)
Bias arising from randomization process	Some concerns	Low risk	Low risk	Low risk	Some concerns
	*Reported in the article “Random assignment was performed by a professional statistician according to pre‐defined randomization tables”*	*Reported in the article “the patient's inclusion in one of the two treatment groups was done according to predefined randomization tables”*	*Reported in the article “Each patient was selected according to predefined randomization tables”*	*Reported in the article “One independent investigator independent from those carrying out the screening performed the randomization sequence using random block sizes that were stratified according to tobacco. Allocation concealment was kept using opaque‐sealed envelopes”*	*Reported in the article “Sequence of installation was randomly determined using sealed envelopes”*
		“Allocation was implemented by an independent examiner, who received a sealed opaque envelope for each patient treatment”	*“The assignment was performed by an independent examiner, who received a sealed opaque envelope”*		
Bias due to deviations from intended interventions	Low risk	Low risk	Low risk	Low risk	Low risk
	*No deviations arose because of trial context*	*No deviations arose because of trial context*	*No deviations arose because of trial context*	*No deviations arose because of trial context*	*No deviations arose because of trial context*
Bias due to missing outcome data	Low risk	Low risk	Low risk	Some concerns	Low risk
	*All data presented*	*Number and reasons for withdrawals were reported. It does not seem that the lost data had affected the results*	*All data presented*	*Number and reasons for withdrawals were reported. The lost data might have affected the results*	*All data presented*
Bias in measurement of the outcome	Low risk	Low risk	Low risk	Low risk	Low risk
	*Blinding was not possible due to the use of two different types of implants. It is likely that assessment was not influenced by knowledge of intervention*	*Blinding was not possible due to the use of two different types of implants. It is likely that assessment was not influenced by knowledge of intervention*	*Blinding was not possible due to the use of two different types of implants. It is likely that assessment was not influenced by knowledge of intervention*	*Blinding was not possible due to the use of two different types of implants. It is likely that assessment was not influenced by knowledge of intervention*	*Blinding was not possible due to the use of two different types of implants. It is likely that assessment was not influenced by knowledge of intervention*
Bias in selection of the reported result	Low risk	Low risk	Low risk	Low risk	Low risk
	*All outcomes appear to be detected*	*All outcomes appear to be detected*	*All outcomes appear to be detected*	*All outcomes appear to be detected*	*All outcomes appear to be detected*
Overall risk of bias	Some concerns	Low risk	Low risk	Some concerns	Some concerns

Three studies (Lago et al. 2018, 2019; Sanz‐Martín et al. 2017) have reported on the methods of randomization and adequately described the allocation concealment and therefore were judged to be at low risk of bias for this domain. None of the studies reported on masking the data assessors. However, blinding was not possible due to different implant designs (tissue level vs. bone level). In the area of incomplete or missing outcome data, four studies (Fernández‐Formoso et al. 2012; Lago et al. 2018, 2019; Vianna et al. 2018) demonstrated a low risk of bias, while one study (Sanz‐Martín et al. 2017) raised some concerns because of its very high attrition rate. None of the included studies showed deviations from the planned interventions and every outcome seemed to be identified. Therefore, they were all assigned a low‐risk rating for those domains. Four studies (Lago et al. 2018, 2019; Sanz‐Martín et al. 2017; Vianna et al. 2018) reported on sample size calculation. There was no information about any attempt to register the trials before their start.

### 3.6 Effects of Interventions

The current review included 241 participants with 501 dental implants in total. Of these, 149 participants received tissue level implants, and the remaining participants received bone level implants (Table 4). The data were presented at the participant and implant level.

**Table 4:** Summary of findings: Tissue level versus bone level implants.

Outcome	Number of studies	Relative effect (95% CI)	Anticipated absolute effects* (95% CI)		Certainty of the evidence (GRADE)†
			TLI	BLI	
Changes peri‐implantitis rate	5 studies	RR 0.59 (0.14–2.48)	1000 per 1000	590 greater per 1000	⊕⊕⊝⊝
				140 greater to 2480 greater	Low^a^,^b^
Changes in marginal bone level at 12 months (mm)	4 studies	Not estimable	The mean ranged across control groups from −0.15 to 0.38	MD 0.15 higher	⊕⊝⊝⊝
				(0.11 lower to 0.40 higher)	Very low^a^,^b^,^c^
Changes in probing pocket depths at 12 months (mm)	3 studies	Not estimable	The mean ranged across control groups from −0.29 to −0.10	MD 0.08 lower	⊕⊕⊝⊝
				(0.32 lower to 0.17 higher)	Low^a^,^c^
Changes in probing pocket depths at 24 months (mm)	2 studies	Not estimable	The mean ranged across control groups from 6.30 to 7.60	MD 0.40 lower	⊕⊕⊝⊝
				(0.94 lower to 0.14 higher)	Low^a^,^c^
Implant failure rate	5 studies	RR 0.59 (0.07–4.69)	1000 per 1000	590 greater per 1000	⊕⊕⊝⊝
				(70 greater to 4690 greater)	Low^a^,^b^

*Note:* GRADE working group grades of evidence

High certainty: We are very confident that the true effect lies close to that of the estimate of the effect.

Moderate certainty: We are moderately confident in the effect estimate: the true effect is likely to be close to the estimate of the effect, but there is a possibility that it is substantially different.

Low certainty: Our confidence in the effect estimate is limited: the true effect may be substantially different from the estimate of the effect.

Very low certainty: We have very little confidence in the effect estimate: the true effect is likely to be substantially different from the estimate of effect.

Abbreviations: BLI, bone level implants; CI, confidence interval; MD, mean difference; RR, risk ratio; TLI, tissue level implants.

^a^ Downgraded one level due to risk of bias: Inadequate concealment of allocation.

^b^ Downgraded one level due to risk of bias: Incomplete accounting of participants and outcome events.

^c^ Downgraded one level due to inconsistency: Heterogeneity was detected.

* The risk in the intervention group (and its 95% CI) is based on the assumed risk in the comparison group and the relative effect of the intervention (and its 95% CI).

^†^ None of the studies suffered from indirectness or detected publication bias.

#### 3.6.1 Peri‐Implantitis Rate

The rate of peri‐implantitis was reported in all studies (Fernández‐Formoso et al. 2012; Lago et al. 2018, 2019; Sanz‐Martín et al. 2017; Vianna et al. 2018). Overall, peri‐implantitis occurred in two tissue level implants and four bone level implants. The difference was not statistically significant (relative risk [RR] 0.59; 95% CI 0.14–2.48; *p* = 0.47; Figure 3a). Heterogeneity was not observed (*χ* ^2^ = 0.42, df = 2 [*p* = 0.81]; *I* ^2^ = 0%).

Figure 3Comparison: Tissue level versus bone level implants. Primary outcome: (a) Peri‐implantitis rate. Secondary outcomes: (b) Changes in marginal bone level at 12 months, (c) changes in marginal bone level at 24 months, (d) changes in marginal bone level at 36 months, (e) changes in marginal bone level at 60 months, (f) changes in probing pocket depth at 12 months, (g) changes in probing pocket depth at 24 months, and (h) implant failure rate. CI, confidence interval; IV, inverse variance; SE, standard error; z, z test; τ, Kendall tau.

**Figure:**
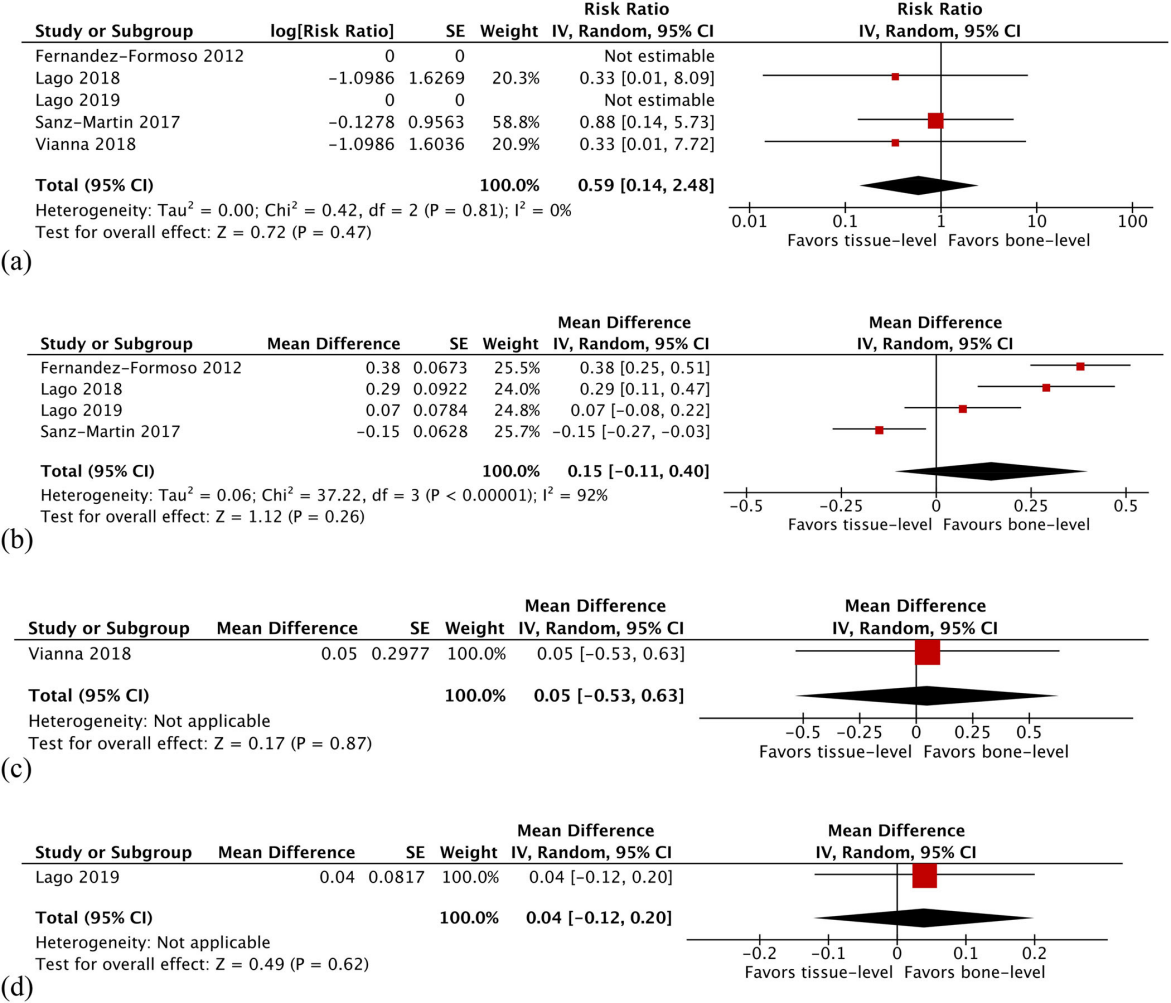

**Figure:**
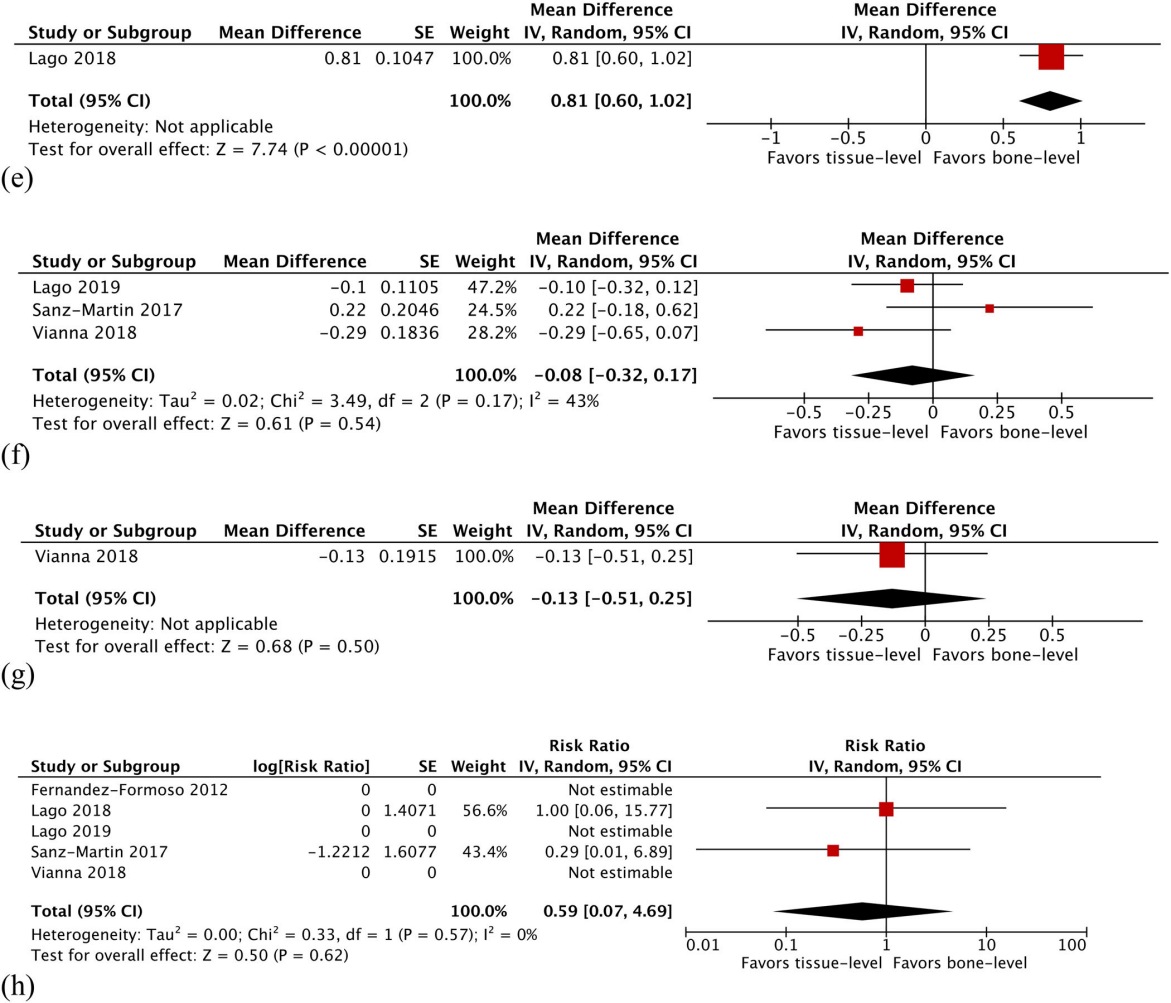

#### 3.6.2 Changes in Marginal Bone Level

All the included studies (Fernández‐Formoso et al. 2012; Lago et al. 2018, 2019; Sanz‐Martín et al. 2017; Vianna et al. 2018) described the changes in marginal bone level at different time intervals. At 12 months, the overall meta‐analysis showed that tissue level implants had greater changes in marginal bone level compared with bone level implants but without any statistically significant difference (MD 0.15; 95% CI −0.11 to 0.40; *p* = 0.26; Figure 3b). Significant heterogeneity was detected (*χ* ^2^ = 37.2, df = 3 (*p* < 0.0001); *I* ^2^ = 92%). The differences between the two groups were also not significant at 24 (MD 0.05; 95% CI −0.53 to 0.63; *p* = 0.87; Figure 3c), and 36 months (MD 0.04; 95% CI −0.12 to 0.20; *p* = 0.62; Figure 3d). The changes in marginal bone level at 60 months were in favor of bone level implants but the meta‐analysis was only based on one study (MD 0.81; 95% CI 0.60–1.02; *p* < 0.0001; Figure 3e).

#### 3.6.3 Changes in Probing Pocket Depth

The variations in the probing pocket depth were reported in three studies (Lago et al. 2019; Sanz‐Martín et al. 2017; Vianna et al. 2018). The tissue level implant group had more positive changes than the bone level implant group, however, there was no statistically significant difference between the two groups at 12 months (MD −0.08; 95% CI −0.32 to 0.17; *p* = 0.54; Figure 3f). There was evidence of moderate heterogeneity (*χ* ^2^ = 3.49, df = 2 [*p* = 0.17]; *I* ^2^ = 43%). The difference between the two groups was also insignificant at 24 months (MD −0.13; 95% CI −0.51 to 0.25; *p* = 0.50; Figure 3g).

#### 3.6.4 Implant Failure Rate

The implant failure rate was recorded in all studies. One implant failed in the tissue level implant group, while two failed in the bone level implant group. There was no statistically significant difference between the two groups (RR 0.59; 95% CI 0.07–4.69; *p* = 0.62; Figure 3h). There was no evidence of heterogeneity (*χ* ^2^ = 0.33, df = 1 [*p* = 0.57]; *I* ^2^ = 0%).

#### 3.6.5 Sensitivity Analyses

The leave‐one study‐out sensitivity analysis showed that the overall effect‐size estimate for peri‐implantitis rate (pooled RR range, 0.33–0.69) or heterogeneity (*I* ^2^ 0%) was not significantly changed when excluding each one of the included studies. Additionally, the difference between the two groups has been negligible, indicating that none of the studies was an outlier or had a disproportionate impact on the overall estimate of peri‐implantitis rate (Table 5).

**Table 5:** Leave‐one study‐out sensitivity analysis: Changes in peri‐implantitis rate.

Removed study	Overall RR (95% CI)	*p* value	Heterogeneity
Lago et al. (2018)	0.68 (0.14, 3.14)	*p* = 0.64	*p* = 0.60; *I* ^2^ = 0%
Sanz‐Martín et al. (2017)	0.33 (0.04, 3.13)	*p* = 0.34	P = 1.00; *I* ^2^ = 0%
Vianna et al. (2018)	0.69 (0.14, 3.45)	*p* = 0.65	*p* = 0.61; *I* ^2^ = 0%

Abbreviations: CI, confidence interval; RR, risk ratio.

## 4 Discussion

### 4.1 Summary of Main Results

The present systematic review compared tissue level to bone level dental implants in terms of peri‐implantitis rate, marginal bone level, probing pocket depth, and implant failure rate. Tissue level implants had lower rates of peri‐implantitis and implant failure and less changes in probing pocket depths at 12 and 24 months compared to bone level implants. The difference between the two groups was not statistically significant. Greater changes in marginal bone level were observed around tissue level implants compared to bone‐level ones but the differences at different time points were not significant except at 60 months.

### 4.2 Quality of Evidence and Limitations

In the present systematic review, we included only randomized controlled trials with strict selection criteria to improve the quality of the search and minimize expected heterogeneity. In this context, the comparison was limited to studies that only included implants of similar surface characteristics to minimize confounding factors. For example: Studies comparing machined and moderately roughened implant surfaces were excluded. A quantitative analysis, based on studies with moderate to high quality, was presented in this review. Several meta‐analyses revealed moderate to substantial heterogeneity amongst the included studies, specifically with regard to changes in marginal bone level and probing pocket depths. Although similar implant systems and identical implant placement and loading protocols were employed, possible sources of heterogeneity could still exist due to the inclusion of anterior and posterior sites as well as the absence of a precise case definition of peri‐implantitis. Only one study (Sanz‐Martín et al. 2017) defined peri‐implantitis using the standards established by the 7th European Workshop in Periodontology (Lang et al. 2011), while others (Fernández‐Formoso et al. 2012; Lago et al. 2018, 2019; Vianna et al. 2018) made no reference to any criteria.

Three randomized controlled trials (Lago et al. 2018, 2019; Sanz‐Martín et al. 2017) provided adequate descriptions of the randomization process and allocation concealment and were rated as low risk for this domain. The remaining two studies (Fernández‐Formoso et al. 2012; Vianna et al. 2018) were rated as having some concerns since there was insufficient information on the randomization and allocation concealment. As blinding of the clinical and radiographic parameters was not feasible, studies were rated as having a low risk of bias in the measurement of outcomes. One study (Sanz‐Martín et al. 2017) raised some concerns in terms of the number and reasons for withdrawals, but the other four (Fernández‐Formoso et al. 2012; Lago et al. 2018, 2019; Vianna et al. 2018) were considered to have a low risk of bias in the attrition or reporting domains as the reported dropouts did not appear to have an impact on the overall effect estimate.

Despite the limited number of included studies, the precision of treatment effects has been improved by the use of similar methodologies in assessing changes in soft and hard tissues across the included studies. These approaches included the use of standardized periapical radiograph and manual periodontal probe. In addition, three randomized controlled trials (Lago et al. 2018, 2019; Vianna et al. 2018) indicated adherence to the CONSORT criteria although the registration status of the included trials was not clear. Overall, two studies (Lago et al. 2018, 2019) had a low risk of bias for all domains, while three studies (Fernández‐Formoso et al. 2012; Sanz‐Martín et al. 2017; Vianna et al. 2018) had only one domain that raised concerns and a low risk of bias in the other domains.

This review has several important limitations that should be considered when interpreting its findings. First, the number of eligible studies was small, which may restrict the statistical power and the ability to draw robust conclusions. Second, there was moderate to substantial heterogeneity across the included studies, which may further complicate direct comparisons. Third, the lack of a consistent and standardized definition of peri‐implantitis across studies may limit the reliability of pooled peri‐implant disease outcomes. Fourth, most studies had relatively short follow‐up durations, preventing assessment of long‐term implant performance and complications. Additionally, trial registration was often unclear or absent, raising concerns regarding selective reporting and methodological transparency. Overall, the certainty of evidence was rated as low to very low, and these factors collectively limit the generalizability of the findings. Consequently, care should be exercised when interpreting the results of this systematic review.

### 4.3 Applicability of Evidence

The present systematic review has shown that the use of tissue level and bone level implants is associated with comparable rates of peri‐implantitis and implant failure. Clinically acceptable changes in probing pocket depth and marginal bone level were noted in all studies. Peri‐implantitis rates at the implant level ranged from 0% to 9%, which is significantly lower than previously reported (Atieh et al. 2013; Derks et al. 2016). The reduced incidence of peri‐implantitis in this review may be explained by the relatively short follow‐up periods of the included studies.

It is worth noting that the lack of statistical significance was evident amongst the majority of the evaluated outcomes except for changes in marginal bone level at 60 months where differences were statistically significant and in favor of bone‐level implants. The clinical relevance of such difference remains questionable considering that the analysis was based on one study (Lago et al. 2018). It should also be noted that the clinical criteria for peri‐implantitis were only reported in one short‐term study (Sanz‐Martín et al. 2017). Thus, more data are still required to determine the long‐term effect of tissue level and bone level implants on the incidence of peri‐implantitis. In addition, the cost‐effective benefits of using tissue level implants in comparison to bone level implants remain to be determined as long‐term data on this outcome are lacking. These questions are clinically relevant for informed decision making particularly when the financial burden of management of peri‐implantitis is considered.

One of the strengths of this review is the relative homogeneity in implant design features as all the included studies compared platform‐matched tissue level implants to platform‐switched bone‐level implants, both with moderately rough implant surfaces. This has eliminated multiple sources of heterogeneity often encountered in systematic reviews on the prevalence of peri‐implantitis (Atieh et al. 2013; Diaz et al. 2022; Lee et al. 2017). Nevertheless, there was low certainty of evidence that tissue level implants reduced the rates of peri‐implantitis and implant failure and minimized changes in probing pocket depth at 12 and 24 months, while there was very low certainty of evidence that bone‐level implants minimized changes in marginal bone level at 12 months.

### 4.4 Agreements and Disagreements With Other Systematic Reviews

Several systematic reviews have compared the clinical and radiographic outcomes of tissue level to bone level implants (Cosola et al. 2020; Liu et al. 2021; Mortazavi et al. 2021; Vouros et al. 2012). These reviews included implants with different designs and surface characteristics (e.g., machined and roughened surfaces), complicating outcome comparisons and limiting reliable interpretation of the meta‐analysis results. Nevertheless, and in accordance with the findings of the present review, favorable changes in marginal bone levels amongst bone level implants were demonstrated (Liu et al. 2021; Vouros et al. 2012). Moreover, the risk of peri‐implantitis was also comparable between tissue level and bone level implants (Liu et al. 2021), a finding that has also been demonstrated in the present review. The results of the present review have shown comparable clinical and radiographic outcomes of tissue level and bone level implants of similar designs and surface characteristics. It could be, therefore, inferred that the selection between tissue level and bone level implants largely depend on clinician preference, patient esthetic demands, and related site‐specific characteristics.

Future studies on tissue level and bone level implants should focus on investigating the clinical impact of patient‐ and prosthetic‐related factors on the long‐term treatment outcomes, cost‐effectiveness, and patient‐ and clinician‐reported outcomes.

## 5 Conclusions

Tissue level and bone level implants have comparable survival rates and risk of peri‐implantitis within 1 to 5 years of observation. Additionally, no significant differences in probing pocket depths and marginal bone level changes were observed. More information from long‐term, meticulously planned randomized controlled trials that adhere to CONSORT principles is still needed.

## Author Contributions

**Momen A. Atieh:** concept/design, data collection, data analysis/interpretation, drafting article, critical revision of article, approval of article. **Maanas Shah:** data analysis/interpretation, critical revision of article, approval of article. **Abeer Hakam:** critical revision of article, approval of article. **Ahmad Aid:** data collection, data analysis/interpretation, critical revision of article, approval of article. **Andrew Tawse‐Smith:** critical revision of article, approval of article. **Nabeel H. M. Alsabeeha:** critical revision of article, approval of article.

## Conflicts of Interest

The authors declare no conflicts of interest.

## Data Availability

The data that support the findings of this study are available on the request from the corresponding author. The data are not publicly available due to privacy or ethical approval.
